# Diagnostic Value of a Modified Version of Wilson’s Diagnostic Score in Pediatrics

**Published:** 2020

**Authors:** S. Sajedianfard, M. Ataollahi, S. M. Dehghani

**Affiliations:** 1 *Department of Pediatrics, Shiraz University of Medical Sciences, Shiraz, Iran*; 2 *astroenterohepatology Research Center, Shiraz University of Medical Sciences, Shiraz, Iran*; 3 *Organ Transplantation Center, Nemazee Hospital, Shiraz University of Medical Sciences, Shiraz, Iran*

**Keywords:** Hepatolenticular degeneration, Wilson’s disease, Ceruloplasmin, Liver transplantation

## Abstract

Background:

Wilson’s disease (WD) is an autosomal-recessive hereditary liver disease affecting copper metabolism.

**Objective:**

To test the diagnostic value of a questionnaire for the diagnosis of WD in pediatrics age group.

**Methods:**

70 children with biopsy-proven diagnosis of WD and 70 without WD were included in the study. A modified questionnaire with 4 items was used for the diagnosis of WD. The results were then compared to the definite diagnosis made by pathology (the gold standard test).

**Results:**

The median (IQR) modified score in those with WD was 4 (4–5), significantly (p<0.001) higher than that calculated for the comparison group, which was 0 (0–1). The most appropriate cut-off value for the score was 2.5, corresponding to a sensitivity and specificity of 100%, and 98.6%, respectively. Using this cut-off value to classify 20 children with and without WD who underwent liver transplantation resulted in an accuracy of 100%.

**Conclusion:**

The modified scoring system is a sensitive and specific diagnostic tool for the diagnosis of WD in children. This is especially important in regions with limited access to specific laboratory tests for the diagnosis of WD.

## INTRODUCTION

Wilson’s disease (WD) is an autosomal-recessive hereditary liver disease affecting copper metabolism. The prevalence of the disease ranged from 1.5 to 1.81 per 100,000 around the world [1, 2]. WD usually presents with hepatomegaly and acute or subacute hepatitis in children aged three years or more [3]. It is one of the common causes of liver failure and liver transplantation around the globe [4-8]. A recent multicenter study revealed that WD is the cause of 14% of pediatrics liver transplantations in Iran [9]. Therefore, early diagnosis of the disease would be beneficial and may even prevent progress of the disease into hepatic failure.

Several diagnostic means have so far been used for the diagnosis of WD [10-13]. These include measurement of the serum ceruloplasmin and 24-hour urine copper, and presence of Kayser-Fleischer (KF) ring, among other tests [14, 15]. Nonetheless, these diagnostic tests are not readily available in all centers, particularly in developing countries. Several questioners are available to determine the likelihood of presence of the disease based on certain clinical and available laboratory data. For example, a Wilson diagnostic score has long been used for screening of patients with suspicious WD [16, 17]. The scoring system includes eight items, *i.e.*, presence of KF rings (maximum of 2 points), neuropsychiatric symptoms (maximum of 2 points), rhodanine-positive hepatocytes (maximal 1 points), disease-causing mutations (maximum of 4 points), and Coombs-negative hemolytic anemia (maximal of 1 point), and measurement of 24-hour urinary copper (maximum of 2 points), liver copper (maximum of 2 points), and serum ceruloplasmin (maximum of 2 points), with the higher scores indicating higher probabilities of WD. However, quantitative measurement of liver copper, identification of rhodanine-positive hepatocytes, and detection of disease-causing mutations are not available in all centers in developing countries. We, therefore, conducted this study to determine if a modified version of the questionnaire, after exclusion of these items, could still be efficiently used for the diagnosis of WD in our setting in Iran.

## MATERIALS AND METHODS

There were two groups of 70 biopsy-proven patients with WD and 70 without WD who were followed for other conditions in the Pediatric Hepatology Clinic affiliated to Shiraz University of Medical Sciences between 2010 and 2018. All children aged <18 years at presentation. The gold-standard test used for the diagnosis of WD was liver biopsy. All patients in the WD group had also clear clinical presentations of the disease. 

Variables studied included age, sex, presence of KF ring, serum ceruloplasmin level, 24-hour urine copper concentration, presence of neuropsychiatric symptoms, and presence of Coombs-negative hemolytic anemia. Those with prior liver transplantation were excluded from the study.

Modified Scoring System

After exclusion of the three items not available in our center—quantitative measure of liver copper, identification of rhodanine-positive hepatocytes, and detection of disease-causing mutations—from the original questionnaire, we came to a modified scoring system consisting of five items. The total score for each participant was then calculated by adding up the score given to each item in the modified questionnaire.

Ethics

The study protocol was approved by the Ethics Committee of Shiraz University of Medical Sciences. The data were retrieved from the participants’ charts; no one could be identified based on the data.

Statistical Analysis

SPSS^®^ for Windows^®^ ver 20 (SPSS Inc., IL, USA) was used for data analysis. One-sample Kolmogorov-Smirnov test was used to assess the normality of the distribution of continuous variables. Normally distributed variables were presented as mean±SD and compared with parametric statistical tests; otherwise, they were presented as median (IQR) and compared with non-parametric tests [18]. A binary logistic regression analysis (backward stepwise: conditional) was used to determine the independent predictors of WD. The status of WD was considered the dependent variable and all the five items left in the modified questionnaire were considered independent variables.

Receiver operating characteristic (ROC) curve analysis was used to determine the most appropriate cut-off value for the modified score for the diagnosis of WD [19]. The value was defined as the point corresponding to the maximum Youden’s index. Area under the ROC curve was calculated. Sensitivity, specificity, positive and negative predictive values, and positive and negative likelihood ratios, as well as the number-needed-to-misdiagnose (NNM) were also calculated [20]. A p value <0.05 was considered statistically significant.

Test Groups

To test the validity of the new score, we applied the scoring system to classify 10 children with and 10 without WD, chosen at random from those children who underwent liver transplantation during the previous two years in Shiraz Organ Transplantation Center.

## RESULTS

There were 70 (48 [69%] male and 22 [31%] female) patients with definite diagnosis of WD. The mean±SD age of patients was 10±4 years; the youngest was three years old. There were 43 (61%) boys and 27 (39%) girls in the comparison group; the mean±SD age was 12±4 years.

The logistic regression analysis revealed that “neuropsychiatric symptoms,” an item from the five items left in the modified questionnaire, had no independent discriminating value for the diagnosis of WD. The item was thus removed from the questionnaire. The four items left in the final version of the questionnaire along with the scores assigned to each item, are presented in Table 1.

**Table 1:** The final version of the scoring system used

Items	Score		
	0	1	2
Kayser-Fleischer rings	Absent	—	Present
Coombs-negative hemolytic anemia + ↑ Serum copper	Absent	Present	—
Urinary copper (in the absence of acute hepatitis)	Normal	1-2 × ULN	>2 × ULN, or normal but >5 × ULN 1 day after challenge
Serum ceruloplasmin	> 0.2 g/L	0.1–0.2 g/L	< 0.1 g/L

**Figure 1:**
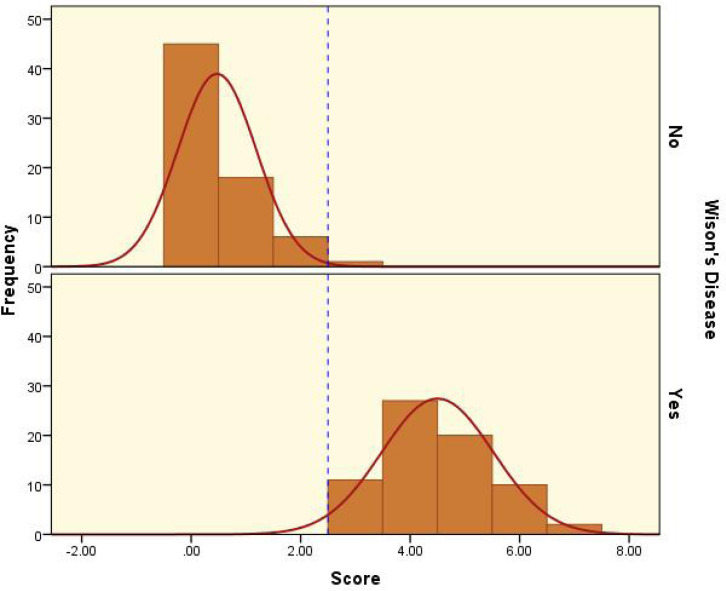
Distribution of the modified score in those with and without Wilson’s disease. The blue dashed line represents the set cut-off value of 2.5

**Figure 2:**
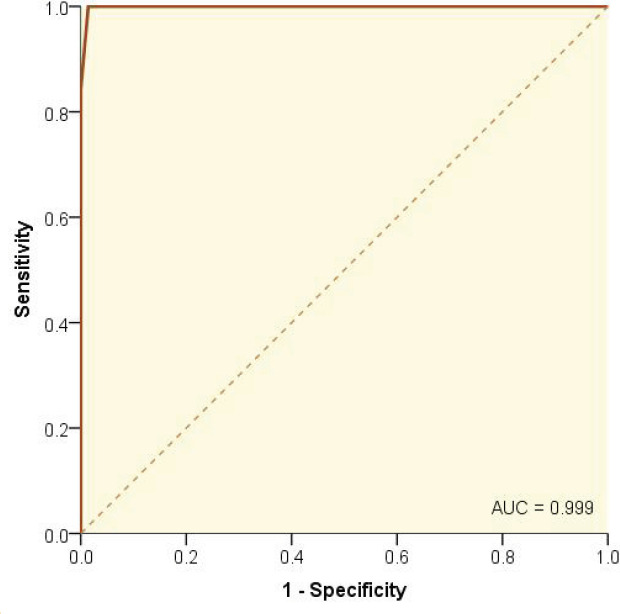
ROC curve indicating an almost perfect discriminating ability of the score

The median (IQR) modified score in patients with WD was 4 (4–5). It was significantly (p<0.001) higher than that calculated for the comparison group, which was 0 (0–1) (Fig 1). The most appropriate cut-off value for the score was 2.5, corresponding to a sensitivity and specificity of 100%, and 98.6%, respectively (Fig 2); it was corresponding to positive and negative likelihood ratios of 70 and 0, respectively. Considering estimated prevalence rates of WD of 1%, 5%, and 10% in the office of a general practitioner, office of a general pediatrician, and office of a pediatrician with specialty in hepatology and gastrointestinal disorders, the positive and negative predictive values, and NNM were calculated (Table 2).

**Figure 3:**
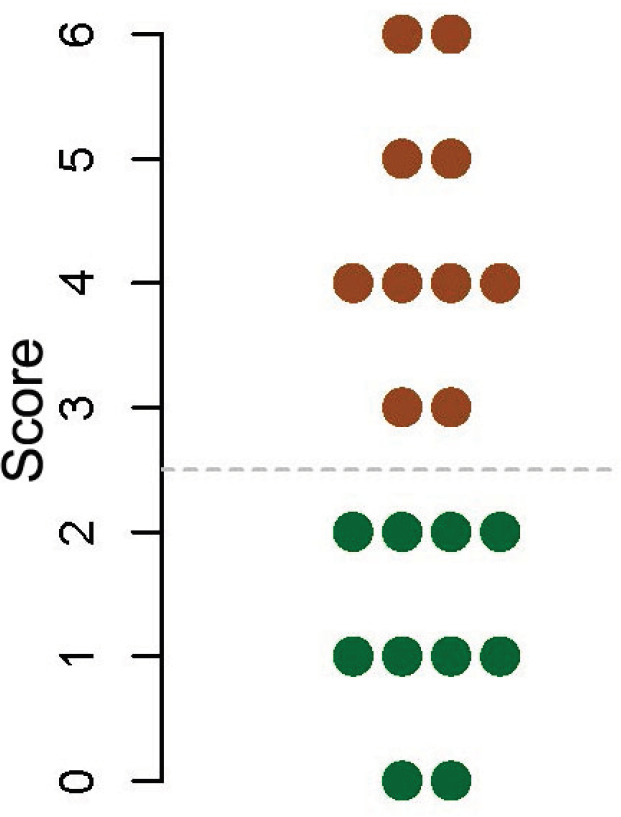
Scores calculated for the test group: 10 with WD (solid brown circles) and 10 without WD (solid green circles). The dashed gray line represents the set cut-off value

The median score calculated for 10 children who underwent liver transplantation during the previous 2 years because of WD, was 4 (range: 3–6), all scores exceeded the set cut-off value of 2.5; the values for 10 children who underwent liver transplantation during the same period for other causes were 1 (range: 0–2), all scores <2.5. This translates to an accuracy of 100%—no case was misclassified (Fig 3).

## DISCUSSION

We found that the modified scoring system had an almost perfect discriminating ability for the diagnosis of WD. It has high sensitivity and specificity of 100% and 98.6%. This high sensitivity means there was no false-negative result, hence, an NPV of 100%. Anyone with a score <2.5 did not have WD. As expected, the PPV increased as the prior probability (prevalence) of the disease increased from more than 40% in the office of a general practitioner (an approximate prevalence of 1%) to 89% in the office of a pediatrician with specialty in hepatology and gastrointestinal disorders (assuming an estimated WD prevalence of 10%). These translated to an NNM ranging from 71 for a general practitioner to 78 for a pediatrician with the specialty. An NNM of 78 means that out of 78 children screened with the modified four-item scoring system (Table 1), only one would be misdiagnosed (either false-negative or false-positive result). Since we did not have any false-negative results, the one out of 78 children misdiagnosed should have a false-positive result. This child should undergo further evaluations. The cut-off value of 2.5 was associated with the maximum Youden’s index; it also provided the maximum diagnostic yield [21]. All these indices show that the new scoring system has indeed a good diagnostic value. This is also clear from the measured area under the ROC curve of almost 1 (Fig 2).

Use of questionnaires for screening patients is not new. For example, other questionnaires have been used in different populations for other gastrointestinal diseases such as autoimmune hepatitis [22-24]. Our scoring system, although not significantly, had a better diagnostic yield compared to most of them. As an example, a questionnaire developed for the diagnosis of autoimmune hepatitis had a sensitivity and specificity of 95.8%, and 100%, respectively [22].

The “neuropsychiatry component,” one of the items ultimately omitted from our five-item questionnaire for lack of significant contribution to the diagnostic yield of the test, would probably have similar distributions in both those with and without WD. That would be attributed to the fact that neuropsychiatry components can also be seen in other gastrointestinal disorders.

Our study had some limitations. In our study, we calculated the score using the weight for each item exactly as it was used in the original questionnaire. However, the four components left in the final scoring system (Table 1) would result in a better discrimination of those with and without WD, if other weights were used. It is, therefore, suggested to reassess the weights of each item in the modifying scoring system. Another limitation was that the scoring system was applied only to patients visited in a single center. Thus, it is of paramount importance to reassess the yield of this scoring system in other centers to examine its robustness.

In conclusion, the four-item scoring system presented in this article is an almost perfect diagnostic/screening tool for the diagnosis of WD in pediatrics age group.
